# Evaluation of Mineral Content in Preterm Human Milk and Infant Formulas in Qatar: Assessing Compliance With Dietary Recommendations and Label Accuracy

**DOI:** 10.1002/fsn3.70828

**Published:** 2025-11-10

**Authors:** Zainab Haji, Rana Mohammed, Hanan Fardan, Najmeh Vatankhah, Shrooq Ismail, Maya Bassil, Zumin Shi, Mohammad Ibrahim Ahmad Ibrahim, Grace Attieh, Nader Al‐Dewik, Layal Karam

**Affiliations:** ^1^ Department of Nutrition Sciences, College of Health Sciences, QU Health Qatar University Doha Qatar; ^2^ Central Laboratories Unit Qatar University Doha Qatar; ^3^ Department of Neonatology, Neonatal Intensive Care Unit, Newborn Screening Unit Women's Wellness and Research Center, Hamad Medical Corporation Doha Qatar; ^4^ Genomics and Precision Medicine (GPM), College of Health & Life Science (CHLS) Hamad Bin Khalifa University (HBKU) Doha Qatar; ^5^ Department of Research Women's Wellness and Research Center, Hamad Medical Corporation Doha Qatar; ^6^ Department of Translational & Precision Medicine Research Facility Women's Wellness & Research Center (WWRC), Hamad Medical Corporation (HMC) Doha Qatar

**Keywords:** copper, human milk, infant formula, iron, manganese, preterm, Qatar, trace minerals, zinc

## Abstract

Preterm infants have higher energy and nutrient needs compared to term infants, with human milk recommended as the primary feeding choice and infant formula as the secondary option. This study aimed to evaluate the concentration of essential trace minerals (manganese, copper, iron, and zinc) in preterm human milk and infant formulas in Qatar, and assess their nutrition label accuracy and compliance with nutritional requirements. Mineral analysis was performed using Inductively Coupled Plasma Mass Spectrometry (ICP‐MS). Samples included 50 liquid human milk samples from lactating mothers of preterm infants, 42 powder infant formulas from local markets and pharmacies, and 10 water samples commonly used in Qatar. All human milk and infant formulas were below the recommended zinc and iron ranges as per the American Society for Parenteral and Enteral Nutrition (ASPEN). Additionally, 96% (48/50) of human milk and 95% (40/42) of infant formulas were below the recommended copper range. Furthermore, 34% (17/50) of human milk samples for manganese were below the recommended range, whereas 86% (36/42) of infant formulas exceeded it. Water samples showed mineral levels below detection limits, and thus had no contribution to mineral levels in reconstituted formulas. Significant differences were found between label and laboratory‐tested values for copper (*p* = 0.0039) and zinc (*p* = 0.0000), with label values higher than laboratory results. No significant differences were observed for manganese (*p* = 0.7564) or iron (*p* = 0.1966). Reconstituted formulas had significantly higher manganese, zinc, and iron laboratory levels (*p* < 0.001) than human milk, whereas copper showed no significant difference (*p* = 0.324). These findings highlight mineral imbalances in both human milk and infant formulas for preterm infants, demonstrating the need for human milk fortifiers, improved nutrient formulation, accurate labeling, and further research to ensure optimal health outcomes.

## Graphical abstract

Zinc and iron concentrations were below ASPEN recommendations in all human milk and formula samples. Copper was below the recommendations in 96% of human milk and 95% of formula, whereas manganese was below recommendations in 34% of human milk and exceeded them in 86% of formula samples. Labels overestimated zinc and copper compared to lab results; no differences were found for manganese or iron. Reconstituted formulas had higher manganese, zinc, and iron than human milk, whereas copper levels were similar.

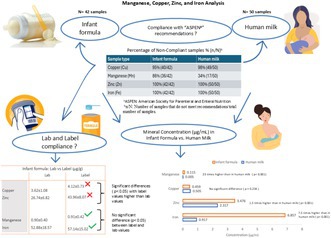

## 1 Introduction

Preterm birth is a serious global public health concern, associated with increased risks of child morbidity, mortality, and long‐term health effects (Morken 2010). With the prevalence of preterm births rising worldwide, a population‐based study for preterm birth in the state of Qatar conducted in 2021 reported about 9% birthrate (Salama et al. 2021). In response to these risks, human milk is recognized as essential due to its rich bioactive components, which promote neonatal organ development, strengthen the immune system, reduce the risk of infections and chronic diseases in infants, and support healthy gut microbiota (Andreas et al. 2015).

Furthermore, human milk enhances neurodevelopmental outcomes, particularly in preterm infants, with benefits increasing proportionally to breastfeeding duration (Rozé et al. 2012; Reniker et al. 2023; Duale et al. 2022). Therefore, the World Health Organization (WHO) recommends exclusive breastfeeding for the first 6 months (WHO 2022).

However, several studies showed that premature infants have a lower likelihood of initiating breastfeeding and sustaining exclusive breastfeeding compared to term infants (Giannì et al. 2016). This disparity stems from challenges such as increased sleepiness, reduced muscle strength, and greater difficulty with latching, sucking, and swallowing (Lapillonne et al. 2013; Meier et al. 2010; Rayfield et al. 2015). Additional barriers, including insufficient breast milk production, inadequate breastfeeding support, societal influences, and workplace constraints (Zhang et al. 2015; Cerniglia et al. 2019), may lead mothers to opt for infant formula as an alternative or supplementary feeding method (Rayfield et al. 2015; Zhang et al. 2015). This trend is reflected in the global infant formula market, which showed 121.5% increase in global infant formula sales of between 2005 and 2019 (Baker et al. 2021).

Minerals play a vital role in growth and brain development, particularly in late pregnancy or after premature birth, which can be met through both infant formula and human milk (Follett et al. 2024). Organizations such as the European Society for Pediatric Gastroenterology, Hepatology, and Nutrition (ESPGHAN) and the American Academy of Pediatrics have established guidelines suggesting that preterm infants may require higher levels of certain nutrients compared to full‐term infants to support their growth and development (American Academy of Pediatrics 2020; Agostoni et al. 2010). Trace minerals, including zinc, copper, iron, and manganese, are essential for normal growth, immune function, and neurological development in preterm infants, along with other important minerals. However, excessive concentrations of trace elements can lead to toxicity depending on the specific mineral and the level and duration of exposure (Hossain et al. 2021; Domellöf 2014).

Studies from various countries including Brazil (de Almeida et al. 2022), United States, and France (Frisbie et al. 2019), USA, UK, and Nigeria (Ikem et al. 2002), Taiwan (Liao et al. 2024), Poland (Lesniewicz et al. 2010), Sweden (Ljung et al. 2011), Japan (Higashi et al. 1988), USA (Griffin et al. 2013), and EU (Pandelova et al. 2012) have investigated trace elements in infant formula. Similarly, numerous studies from Italy (Prohaska et al. 2000), Brazil (de Figueiredo et al. 2009), Korea (Kim et al. 2012), United States (Moran et al. 1983), and Japan (Yamawaki et al. 2005) have also examined trace element content in human milk. Moreover, some studies compared trace element concentrations between human milk and infant formula, revealing higher concentrations of certain elements in infant formula compared to human milk (Rodriguez Rodriguez et al. 2000; Fioravanti et al. 2020; Han et al. 2011; Khaghani et al. 2010; Purkiewicz et al. 2024).

Although some studies from Gulf countries such as Saudi Arabia (Al‐Saleh et al. 2003; Alfaris et al. 2022), Kuwait (Jallad 2024; Al‐Awadi and Srikumar 2000), and the UAE (Abdulrazzaq et al. 2008) have analyzed trace elements in breast milk and infant formulas, they have focused on term infants and general product assessments. No similar research has been conducted in Qatar, nor specifically on preterm infants. Additionally, prior studies have not compared labeled versus lab‐measured mineral content in formulas. This study addresses these gaps by evaluating trace minerals in human milk and infant formula, assessing labeling accuracy, and generating locally relevant data to support region‐specific guidelines and improve preterm infant care.

To address these knowledge gaps, the present study aimed to: (a) analyze and compare the concentrations of four essential minerals (manganese, copper, iron, zinc) in preterm human milk and infant formulas in Qatar, (b) assess whether mineral content in both human milk and infant formula types meets international standards and dietary recommendations, and (c) evaluate the accuracy of mineral content stated on infant formula labels compared to laboratory‐tested concentrations.

## 2 Materials and Methods

### 2.1 Human Milk Samples Collection

Using convenience sampling, human milk samples were collected from mothers of preterm infants (born < 38 weeks gestation) in the Neonatal Intensive Care Unit (NICU) of the Women's Wellness and Research Center at Hamad Medical Corporation (HMC) in Doha, Qatar. Qatar University (QU IRB #: QU‐IRB 133/2024‐EM) and HMC (HMC IRB #: MRC‐01‐22‐231) Institutional Review Boards (IRBs) approved the study. A total of 50 participants were recruited after providing informed consent. The inclusion criteria consisted of mothers who were exclusively breastfeeding a single infant (excluding those having twins or triplets) and who had resided in the designated area for at least the past 5 years. These criteria were created to guarantee a particular study population within specified demographic restrictions and uniform medical environments. Most of the participating mothers (*n* = 43; 86%) were expats, whereas only seven (14%) were locals. At the time of milk collection, mothers were healthy with no comorbidities. Their average age was 31 ± 4 years, with a mean BMI of 22.2 ± 3.1 kg/m^2^ and a mean gestational age at delivery of 31.4 ± 4.4 weeks. Maternal diet and lifestyle were collected as part of the broader study, but they were not included in the current analysis, which focused solely on comparing the mineral composition of preterm human milk and formula milk. Participants were instructed to provide hindmilk after emptying both breasts of their foremilk. Within 2–4 weeks after giving birth, 100 mL of human milk samples was collected from mothers using a breast pump. The samples were kept in analytical‐grade glass containers with Teflon‐lined caps at +4°C while being transported to the lab, and then they were stored at −20°C in the NICU until chemical analysis.

### 2.2 Infant Formula Samples Collection

Different types and brands of infant formula available in Qatar's main pharmacies and supermarkets were screened. Since preterm infants can be fed both preterm‐specific formulas and standard term infant formulas (McGuire et al. 2004; Vass et al. 2023), we included formulas labeled for both preterm infants and term infants aged 0–6 or 0–12 months, as all are suitable for our study population of preterm infants (McGuire et al. 2004; Vass et al. 2023). These term formulas were therefore included in our analysis to reflect real‐life feeding practices.

As a result, 42 infant formula samples from 21 different brands were acquired on two separate manufacturing dates. The market's available samples were divided into two primary categories: term formulas (34 samples from 17 brands) and preterm formulas (eight samples from four brands). Based on their composition and special characteristics, the full‐term formulas were further separated into six subcategories: regular cow's milk (three brands, six samples), lactose‐free cow's milk (two brands, four samples), goat milk (one brand, two samples), anti‐regurgitation (AR) cow's milk (three brands, six samples), colic and constipation cow's milk (five brands, 10 samples), and hypoallergenic cow's milk (three brands, six samples). To avoid any risk of bias, sample collection and laboratory analysis were carried out by separate personnel. Samples were assigned random codes (without any identifying information) immediately after collection to ensure blinded testing.

### 2.3 Water Samples Collection

To simulate standard procedures for making infant formula, 10 samples of bottled water were collected from five popular brands that are sold in Qatar (2 samples for each). By combining the amounts found in the powdered infant formula with the additional water, the mineral concentration was calculated to evaluate the contribution of water to the mineral levels in the reconstituted formula. The manufacturer's instructions on the label were used to calculate the amounts of water and powdered infant formula used in reconstitution. However, the lab analysis using ICP‐MS, as described in the next section, showed that the concentrations of minerals in the water of reconstituted formula fell below the detection limit (< dl), indicating that the water had no significant effect on mineral levels in reconstituted infant formula.

### 2.4 Content of Minerals

Minerals in human milk, infant formula, and water samples were analyzed following the Environmental Protection Agency (EPA) Method 200.8 (Longbottom et al. 1994). Sample digestion was carried out using a MARS 6 microwave digestion system (Xpress, CEM Corporation, USA). Liquid milk samples (~2 g) were mixed in PTFE vessels with 10 mL of nitric acid (normal LR grade; Sigma‐Aldrich) followed by the addition of 2 mL of hydrogen peroxide (35%; Sigma‐Aldrich). Similarly, milk powder samples (~0.5 g) were mixed in PTFE vessels with 6 mL of nitric acid (normal LR grade; Sigma‐Aldrich) and 2 mL of hydrogen peroxide (35%; Sigma‐Aldrich) and left to stand for 15 min before sealing. All vessels were then heated to 200°C for 30 min in the microwave digestion system. After digestion, the samples were transferred to acid‐washed polyethylene tubes and diluted to 50 mL with ultrapure water. The resultant solutions were analyzed for Zn, Mn, Cu, Fe, and Cr using Inductively Coupled Plasma Mass Spectrometry (ICP‐MS) (Perkin Elmer, NexION 300D, USA) with a collision/helium reaction cell system. Data processing was performed using Syngistix software for ICP‐MS. The standard operating conditions for the ICP‐MS included the following: Argon gas (99.998% purity); RF power of 1500 W; spray chamber temperature of 2°C; argon gas flows of 1.3 L/min for the coolant and 0.2 rps for the nebulizer; and a solution uptake rate of 3 mL/min.

### 2.5 Quality Control and Quality Assurance

To ensure the accuracy and precision of results, as well as the recovery rate of the method and instrument, several quality control measures were implemented. These include analysis of certified reference material solutions (Multielement ICP‐MS standard solution from Sigma‐Aldrich), standard reference material (NIST1643F from Sigma‐Aldrich), sample duplicates, and reagent blanks that were analyzed. Mineral concentrations in the samples were quantified using data analysis based on the calibration curve derived from the standards. The limits of detection (LOD) for each mineral, expressed in μg/g, were as follows: Cu (0.00018), Fe (0.00501), Mn (1.8012 × 10^−5^), and Zn (0.00099). The calibration graphs demonstrated excellent correlation coefficients (≥ 0.99), and recovery percentages were strong and ranged from 96% to 100%.

### 2.6 Calculations

To determine the mineral content in reconstituted infant formula, water dilution was calculated based on the manufacturers' instructions provided on the product labels. To calculate the concentrations of minerals in infant formulas and human milk in μg/mL, the density of 1.03 kg/L was used (Ljung et al. 2011; Tanner and Barnett 1986; Yesildemir et al. 2022).

Current guidelines suggest preterm feeding volumes of 140–180 mL/kg/day (up to 200 mL/kg/day); therefore, 150 mL/kg/day was used as a representative average to estimate daily mineral intake from human milk and reconstituted formula (King 2007).

### 2.7 Statistical and Data Analysis

Statistical analyses were performed using the Stata 18 statistical program (StataCorp, College Station, TX, USA, version 18). Continuous variables were presented as means and standard deviations. Pairwise comparisons using the Bonferroni test (with multiple comparisons for *p* value) were performed to compare mineral concentrations between infant formula subcategories and to compare human milk, preterm, and term formulas. One sample *t*‐test was performed to compare mineral concentrations in human milk with all infant formulas and to compare term and preterm formulas. A paired sample *t*‐test was conducted to assess the presence of a significant difference between concentrations of minerals in label and laboratory‐tested milk samples for all infant formula categories. The criterion for statistical significance was set at a *p* value < 0.05.

## 3 Results

The levels and compliance of mineral content in powdered infant formula and human milk samples with the American Society for Parenteral and Enteral Nutrition (A.S.P.E.N) dietary recommendations are shown in Tables 1 and 2 (Vanek et al. 2012).

**TABLE 1:** Compliance of mineral content in both human milk and infant formula samples with the American Society for Parenteral and Enteral Nutrition (A.S.P.E.N) dietary recommendations (Vanek et al. 2012).

Minerals		Do not meet recommendations % (*n*/*N* ^a^)	Above recommendations % (*n*/*N* ^a^)	Below recommendations % (*n*/*N* ^a^)
Human milk^b^	Cu	98% (49/50)	2% (1/50)	96% (48/50)
	Mn	34% (17/50)	0% (0/50)	34% (17/50)
	Zn	100% (50/50)	0% (0/50)	100% (50/50)
	Fe	100% (50/50)	0% (0/50)	100% (50/50)
Infant formula^b^	Cu	95% (40/42)	0% (0/42)	95% (40/42)
	Mn	86% (36/42)	86% (36/42)	0% (0/42)
	Zn	100% (42/42)	0% (0/42)	100% (42/42)
	Fe	100% (42/42)	0% (0/42)	100% (42/42)

^a^ *n*/*N*: number of samples that meet, do not meet, are above, are below recommendations/total number of samples.

^b^ The recommendation for preterm infants set by “A.S.P.E.N” (Vanek et al. 2012) are: copper 12–150 μg/kg/d, zinc 100–3000 μg/kg/d, iron 200–4000 μg/kg/d, manganese 0.7–7.5 μg/kg.

**TABLE 2:** Mean intake (μg/kg/day) ± standard deviation (SD) of minerals in reconstituted infant formulas and human milk.

Category	Subcategory	Brand	*N*	Cu^a^	Mn^a^	Zn^a^	Fe^a^
Term formulas	Cow's milk (Regular)	1	2	70.32 ± 3.98	20.39 ± 0.26	453.12 ± 21.62	1059.12 ± 27.26
		2	2	77.93 ± 3.93	6.83 ± 0.13	551.03 ± 28.92	1205.12 ± 21.97
		3	2	61.91 ± 2.75	24.71 ± 0.59	465.87 ± 52.41	393.26 ± 7.41
	Average^b^		6	70.05 ± 7.69 ^a^	17.31 ± 8.35 ^a^	490.00 ± 55.47^a^	885.83 ± 387.42^a^
	Cow's milk anti‐regurgitation (AR)	1	2	70.44 ± 2.51	32.82 ± 0.32	517.61 ± 20.35	1034.48 ± 6.40
		2	2	80.18 ± 13.61	10.31 ± 0.02	526.98 ± 95.94	1363.20 ± 39.67
		3	2	75.06 ± 0.60	23.71 ± 0.46	756.01 ± 20.74	1070.04 ± 76.95
	Average^b^		6	75.23 ± 7.58 ^a^	22.28 ± 10.13^a^	600.20 ± 129^a^	1155.91 ± 165.96 ^a^
	Cow's milk colic and constipation	1	2	73.64 ± 1.54	6.72 ± 0.14	537.87 ± 28.24	1017.96 ± 67.84
		2	2	57.66 ± 2.73	12.66 ± 0.90	485.75 ± 38.18	856.43 ± 33.26
		3	2	75.59 ± 0.55	22.47 ± 0.53	732.75 ± 25.08	1030.65 ± 2.29
		4	2	67.99 ± 7.81	20.22 ± 0.14	444.84 ± 8.23	1016.98 ± 40.49
		5	2	57.68 ± 14.15	18.67 ± 0.01	386.66 ± 14.45	709.08 ± 51.97
	Average^b^		10	66.51 ± 9.75 ^a^	16.15 ± 6.05^a^	517.57 ± 126.26^a^	926.22 ± 137.12 ^a^
	Cow's milk (Hypoallergenic)	1	2	76.57 ± 6.56	20.47 ± 0.16	416.13 ± 3.52	1119.96 ± 1.47
		2	2	65.55 ± 1.77	20.24 ± 1.59	415.27 ± 8.58	1025.23 ± 39.90
		3	2	54.40 ± 6.71	13.48 ± 3.22	556.29 ± 25.73	1044.98 ± 208.54
	Average^b^		6	65.51 ± 10.79 ^a^	18.06 ± 3.90 ^a^	462.56 ± 73.63^a^	1063.39 ± 104.95 ^a^
	Cow's milk (lactose free)	1	2	71.25 ± 1.97	25.73 ± 1.17	482.88 ± 12.43	1574.91 ± 680.01
		2	2	39.29 ± 51.32	16.61 ± 21.39	295.51 ± 361.43	632.82 ± 637.22
	Average^b^		4	55.27 ± 34.92 ^a^	21.17 ± 13.44^a^	389.20 ± 235.16^a^	1103.87 ± 765.07^a^
	Goat's milk^b^	1	2	76.32 ± 9.58^a^	15.07 ± 1.77 ^a^	536.71 ± 6.53 ^a^	870.73 ± 104.48 ^a^
Average of term formulas			34	67.75 ± 14.52	18.30 ± 7.81	504 ± 130.10	1001.47 ± 314.23
Preterm formulas	Cow's milk (low birth weight and prematurity)	1	2	57.19 ± 8.73	17.66 ± 2.85	500.84 ± 107.58	794.16 ± 116.38
		2	2	68.39 ± 1.15	16.71 ± 1.21	643.29 ± 84.31	956.01 ± 95.23
		3	2	71.93 ± 62.17	8.53 ± 3.64	574.53 ± 379.14	1118.27 ± 1122.89
		4	2	128.77 ± 10.34	9.25 ± 0.26	670.49 ± 21.51	1705.04 ± 117.97
Average of all preterm formulas			8	81.57 ± 38.23 ^a^	13.04 ± 4.81 ^a^	597.29 ± 167.96^a^	1143.37 ± 566.15^a^
Average of all infant formulas			42	70.38 ± 21.20	17.30 ± 7.58	521.45 ± 140.81	1028.50 ± 370.65
Human milk			50	75.76 ± 29.26	0.79 ± 0.38	347.56 ± 167.93	137.59 ± 42.11
*p* ^c^				0.324	< 0.001	< 0.001	< 0.001

^a^ The recommendation for preterm infants set by “A.S.P.E.N” (Abdulrazzaq et al. 2008) is: copper 12–150 μg/kg/day, zinc 100–3000 μg/kg/day, iron 200–4000 μg/kg/day, manganese 0.7–7.5 μg/kg.

^b^ The means sharing the same letter in the same columns are not significantly different at the 5% level.

^c^ Statistically significant difference between all infant formula and human milk samples if *p* value < 0.05.

### 3.1 Laboratory‐Tested Mineral Content in Human Milk

For human milk samples, zinc and iron levels were below the recommended range, with 100% (50/50) of the samples failing to meet the A.S.P.E.N guidelines of 1000–3000 μg kg/day and 2000–4000 μg/kg/day, respectively (Vanek et al. 2012). Similarly, copper levels were below the recommended range of 120–150 μg/kg/day in 96% (48/50) of the samples, whereas only 2% (1/50) exceeded the range and 2% (1/50) met it (Vanek et al. 2012). In contrast, manganese levels showed better compliance with A.S.P.E.N standards, with 66% (33/50) of samples meeting the guideline of 0.7–7.5 μg/kg/day (Vanek et al. 2012). However, 34% (17/50) of the samples were below this range.

### 3.2 Laboratory‐Tested Mineral Content in Infant Formula

In the infant formula samples, zinc and iron levels were below the recommended range, with 100% (42/42) of the samples failing to meet the A.S.P.E.N. guidelines (Vanek et al. 2012) for these nutrients, similar to the results found in the human milk samples (Table 2). For copper, 95% (40/42) of samples were below the recommended range, whereas only 5% (2/42) met the guidelines. These 5% (2/42) of the samples were from brands of the preterm infant formula category, as shown in Table 2. For manganese, 86% (36/42) of the samples exceeded the recommended guideline, and 14% (6/42) of the samples were within the range. These 14% (6/42) of samples were from brands in the colic and constipation, preterm, lactose‐free, and regular cow's milk infant formulas categories, as shown in Table 2. As presented in Table 3, the preterm infant formulas showed higher laboratory values for zinc, copper, and iron compared to term infant formulas except for manganese, with term formula showing higher laboratory values than preterm. For the term formula subcategories, cow's milk (AR) showed the highest laboratory values for zinc, iron and manganese except for copper, where the highest values were reported in goat's milk. Despite this trend, there were no significant differences (*p* > 0.05) in laboratory‐measured zinc, copper, and iron levels between term and preterm formulas, nor across subcategories of term formulas for these minerals. Similarly, manganese levels showed no significant differences across subcategories of term formulas, but a significant difference (*p* = 0.039) was observed between term and preterm formulas.

**TABLE 3:** Mean concentrations (μg/g) ± standard deviation (SD) of minerals in powdered infant formulas compared to the amount reported on the label.

Category	Subcategory	Brand	N	Cu lab	Cu label	Mn lab	Mn label	Zn lab	Zn label	Fe lab	Fe label
Term formulas	Cow's milk (Regular)	1	2	4.10 ± 0.23	4.00	1.19 ± 0.02	1.00	26.40 ± 1.26	45.00	61.70 ± 1.59	54.00
		2	2	4.02 ± 0.20	4.00	0.35 ± 0.01	0.35	28.45 ± 1.49	45.00	62.22 ± 1.13	60.00
		3	2	3.20 ± 0.14	4.00	1.28 ± 0.03	1.30	24.05 ± 2.71	37.00	20.30 ± 0.38	51.90
	Average^a^		6	3.77 ± 0.47^a^	4.00 ± 0.00^a^	0.94 ± 0.46^a^	0.88 ± 0.43^abc^	26.30 ± 2.47^a^	42.33 ± 4.13^a^	48.07 ± 21.53^a^	55.30 ± 3.76^a^
	Cow's milk anti‐regurgitation (AR)	1	2	3.64 ± 0.13	4.00	1.69 ± 0.02	1.55	26.72 ± 1.05	41.00	53.41 ± 0.33	53.00
		2	2	4.14 ± 0.70	4.00	0.53 ± 0.00	1.15	27.21 ± 4.95	53.00	70.38 ± 2.05	52.00
		3	2	3.88 ± 0.03	4.00	1.22 ± 0.02	1.55	39.03 ± 1.07	41.00	55.25 ± 3.97	53.00
	Average^a^		6	3.88 ± 0.39^a^	4.00 ± 0.00^a^	1.13 ± 0.52^a^	1.42 ± 0.21^c^	30.99 ± 6.65^a^	45.00 ± 6.20^a^	59.68 ± 8.57^a^	52.67 ± 0.52^a^
	Cow's milk colic and constipation	1	2	3.80 ± 0.08	4.00	0.35 ± 0.01	0.35	27.77 ± 1.46	45.00	52.56 ± 3.50	52.00
		2	2	2.81 ± 0.13	3.71	0.62 ± 0.04	0.43	23.65 ± 1.86	35.00	41.69 ± 1.62	53.00
		3	2	3.90 ± 0.03	4.00	1.16 ± 0.03	1.18	37.83 ± 1.29	55.00	53.21 ± 0.12	52.00
		4	2	3.96 ± 0.46	3.84	1.18 ± 0.01	0.98	25.91 ± 0.48	41.20	59.24 ± 2.36	62.50
		5	2	3.36 ± 0.82	3.84	1.09 ± 0.00	0.98	22.52 ± 0.84	33.90	41.31 ± 3.03	37.60
	Average^a^		10	3.57 ± 0.56^a^	3.88 ± 0.12^a^	0.88 ± 0.35^a^	0.78 ± 0.35^ab^	27.54 ± 5.83^a^	42.02 ± 8.07^a^	49.60 ± 7.61^a^	51.42 ± 8.39^a^
	Cow's milk (Hypoallergenic)	1	2	3.87 ± 0.33	3.90	1.04 ± 0.01	0.85	21.06 ± 0.18	36.00	56.67 ± 0.07	53.00
		2	2	3.32 ± 0.09	3.80	1.02 ± 0.08	0.94	21.01 ± 0.43	35.00	51.88 ± 2.02	53.00
		3	2	2.65 ± 0.33	2.93	0.66 ± 0.16	0.55	27.08 ± 1.25	37.00	50.87 ± 10.15	39.00
	Average^a^		6	3.28 ± 0.59^a^	3.54 ± 0.48^a^	0.91 ± 0.21^a^	0.78 ± 0.18^ab^	23.05 ± 3.18^a^	36.00 ± 0.89^a^	53.14 ± 5.40^a^	48.33 ± 7.23^a^
	Cow's milk (lactose free)	1	2	3.61 ± 0.10	4.00	1.30 ± 0.06	1.27	24.44 ± 0.63	41.00	79.70 ± 34.41	56.00
		2	2	1.99 ± 2.59	3.85	0.84 ± 1.08	1.60	14.97 ± 18.26	45.00	32.12 ± 32.11	60.00
	Average^a^		4	2.80 ± 1.77^a^	3.93 ± 0.09^a^	1.07 ± 0.68^a^	1.44 ± 0.19^bc^	19.70 ± 11.88^a^	43.00 ± 2.31^a^	55.91 ± 38.64^a^	58.00 ± 2.31^a^
	Goat's milk^a^	1	2	3.94 ± 0.49^a^	3.81 ± 0.00^a^	0.78 ± 0.09^a^	0.64 ± 0.00^abc^	27.71 ± 0.34^a^	35.00 ± 0.00^a^	44.96 ± 5.39^a^	38.00 ± 0.00^a^
Average of term formulas			34	3.54 ± 0.78	3.86 ± 0.25	0.96 ± 0.41	0.98 ± 0.40	26.22 ± 6.56	41.24 ± 6.12	52.20 ± 16.08	51.76 ± 7.11
Preterm formulas	Cow's milk (low birth weight and prematurity)	1	2	2.95 ± 0.45	6.20	0.91 ± 0.15	0.36	25.86 ± 5.55	52.00	41.00 ± 6.01	89
		2	2	3.10 ± 0.05	4.00	0.76 ± 0.05	1.20	29.15 ± 3.82	57.00	43.31 ± 4.31	50
		3	2	3.32 ± 2.87	4.43	0.39 ± 0.17	0.36	26.49 ± 17.48	52.00	51.55 ± 51.77	89
		4	2	6.58 ± 0.53	6.16	0.47 ± 0.01	0.51	34.27 ± 1.10	61.00	87.15 ± 6.03	92
Average of preterm formulas^a^			8	3.99 ± 1.96^a^	5.20 ± 1.06^b^	0.63 ± 0.24^a^	0.61 ± 0.37^a^	28.94 ± 7.93^a^	55.50 ± 4.04^b^	55.75 ± 28.09^a^	80.00 ± 18.56^b^
Average of all infant formulas			42	3.62 ± 1.08	4.12 ± 0.73	0.90 ± 0.40	0.91 ± 0.42	26.74 ± 6.82	43.96 ± 8.07	52.88 ± 18.57	57.14 ± 15.02
*p* ^b^				0.0039		0.7564		0.000		0.1966	
*p* ^c^				0.299	< 0.001	0.039	0.029	0.317	< 0.001	0.632	< 0.001

^a^ The means sharing the same letter in the same column are not significantly different at the 5% level.

^b^ Statistically significant difference between lab and label for the respective minerals: copper, manganese, zinc, and iron if *p* value < 0.05.

^c^ Statistically significant difference between term and preterm infant formula for copper, manganese, zinc, and iron in lab and in label if *p* value < 0.05.

### 3.3 Label‐Reported Mineral Content in Infant Formula

Codex international food standards of infant formula aim to protect infant health and facilitate fair trade by establishing international standards that ensure human milk substitutes, when necessary, are safe, nutritionally adequate, and consistently manufactured. All minerals met Codex standards (Table 4) (WHO, F 2007). For iron, the labeled values met the minimum recommended levels specified by Codex (WHO, F 2007). However, since Codex does not provide a Guidance Upper Level (GUL) or maximum limits for iron, we referred to the maximum levels recommended by EU regulations (European Union 2016). Accordingly, 90% (38/42) of the samples met the label regulations, with 10% (4/42) exceeding them (Table 4). The samples that exceeded the regulations were found in cow's milk (AR) (2/6) and preterm infant formulas (6/8) categories.

**TABLE 4:** Infant formula mineral content as reported on label.

Category	Subcategory	Brand	*N*	Cu label^a^ μg/100 kcal	Mn label^a^ μg/100 kcal	Zn label^a^ mg/100 kcal	Fe label mg/100 kcal
Term formulas	Cow's milk (Regular)	1	2	76.12	19.40	0.67	0.75
		2	2	79.3	6.90	0.90	1.20
		3	2	80	25.04	0.71	1.00
	Cow's milk anti‐regurgitation (AR)	1	2	78.9	6.90	0.90	**1.70** ^**b**^
		2	2	80	22.40	1.03	1.01
		3	2	80	29.87	0.97	1.02
	Cow's milk (colic and constipation)	1	2	78.70	6.90	0.90	1.00
		2	2	77.00	9.00	0.73	1.10
		3	2	78.00	23.01	1.07	1.01
		4	2	75.26	19.21	0.81	1.23
		5	2	76.12	19.40	0.67	0.75
	Cow's milk (Hypoallergenic)	1	2	80	16.63	0.70	1.04
		2	2	70	18.40	0.68	1.04
		3	2	61.68	11.57	0.77	0.82
	Cow's milk (lactose free)	1	2	78	24.90	0.80	1.09
		2	2	75	31.00	0.90	1.20
	Goat's milk^b^	1	2	76.2	12.80	0.70	12.80
Preterm formulas	Cow's milk (low birth weight and prematurity)	1	2	117.8	6.84	0.99	**1.69** ^**b**^
		2	2	80	24.00	1.14	1.00
		3	2	84.17	6.84	0.99	**1.69** ^**b**^
		4	2	117.04	9.69	1.16	**1.75** ^**b**^

^a^ The recommendations for infant formulas set by “Codex” specify a minimum and GUL (guidance upper limit) for the respective minerals per 100 kcal: copper at 35–120 μg, manganese at 1–100 μg, zinc at 0.5–1.5 μg, and iron at 0.45 μg with no recommended GUL (WHO, F 2007).

^b^ Bolded values indicate reported label values that do not meet EU regulations for iron with a minimum of 0.3 and a maximum of 1.3 mg/100 kcal (European Union 2016).

Table 3 shows the mineral levels reported on the labels of the infant formulas. There was a significant difference observed for all minerals between term and preterm infant formulas, with preterm infant formula values being higher than term infant formula for copper (*p* < 0.001), zinc (*p* < 0.001), and iron (p < 0.001). However, the reported levels of manganese in term infant formula were higher than those in preterm infant formula (*p* = 0.029). Among the subcategories of term infant formulas, Cow's milk (AR) showed the highest reported values on the label for copper and zinc; meanwhile, lactose‐free had the highest values for iron. Despite these trends, there were no significant differences (*p* > 0.05) in values reported on labels for the same three minerals. A significantly lower manganese value (*p* < 0.005) was reported on the labels of preterm infant formulas, as compared to specific term infant formulas, including cow's milk (AR) and lactose‐free formulas. A significant (*p* < 0.005) difference in manganese values reported on the labels was detected between the term formulas' subcategories, particularly among cow's milk AR, which had higher reported levels than cow's milk hypoallergenic, and cow's milk Colic and constipation formulas. Long‐term follow‐up studies will help to comprehensively assess the long‐term effects of these differences in manganese levels on infant health outcomes. There was no significant (*p* > 0.005) difference in manganese label values among the other subgroups of term infant formulas.

### 3.4 Comparison Between Laboratory‐Tested and Label‐Reported Mineral Values

For infant formula samples, the mineral content displayed on the label of each brand was also compared to the levels found in tested samples, as shown in Table 3. There was a significant difference between the values reported on the labels and the laboratory values for copper (*p* = 0.0039 < 0.05) and zinc (*p* = 0.0000 < 0.05). The values reported on the labels for copper and zinc in all infant formulas were generally higher than the corresponding laboratory tested values, except for copper in goat milk formula, where the laboratory values exceeded those listed on the label. In contrast, there was no significant difference between values reported on the labels and laboratory values for manganese (*p* = 0.7564) and iron (*p* = 0.1966). For manganese, laboratory values were the same as values reported on the label for all infant formulas except for cow's milk (AR) and lactose‐free samples. However, for iron, the values reported on the label were higher than laboratory values in all infant formulas except for cow's milk (AR), hypoallergenic, and goat's milk.

### 3.5 Comparison Between Laboratory‐Tested Infant Formula and Human Milk

Table 5 provides a comparison between the laboratory reported values of the mineral content of reconstituted infant formulas and human milk. Significantly higher levels of manganese, zinc, and iron (*p* < 0.001) were observed in all reconstituted infant formulas, term and preterm formulas, compared to human milk samples. Manganese, zinc, and iron in reconstituted infant formulas were 23 times, 1.5 times, and 7.5 times higher than in human milk, respectively. In contrast, there was no significant difference in copper levels (*p* = 0.324) between all reconstituted infant formula and human milk samples. In addition, there was no significant difference (*p* > 0.005) observed in the levels of Cu, Zn, and Fe between reconstituted preterm and term formula samples. However, the levels of Mn found in reconstituted term formulas were significantly higher (*p* < 0.005) than those in preterm formula samples.

**TABLE 5:** Mean concentrations (μg/mL) ± standard deviation (SD) of minerals in human milk and reconstituted infant formula.

	N	Cu^a^	Mn^a^	Zn^a^	Fe^a^
Term formulas^b^	34	0.452 ± 0.097^a^	0.122 ± 0.052^a^	3.357 ± 0.867^a^	6.676 ± 2.095^a^
Preterm formulas^b^	8	0.544 ± 0.255^a^	0.087 ± 0.032^b^	3.982 ± 1.120^a^	7.622 ± 3.774^a^
All infant formulas	42	0.469 ± 0.141	0.115 ± 0.051	3.476 ± 0.939	6.857 ± 2.471
Human milk^b^	50	0.505 ± 0.195 ^a^	0.005 ± 0.003^c^	2.317 ± 1.120 ^b^	0.917 ± 0.281^b^
*p* ^c^		0.324	< 0.001	< 0.001	< 0.001

^a^ Infant formula was reconstituted according to the manufacturer's instructions with water presenting no detectable level of minerals.

^b^ The means sharing the same letter in the same column are not significantly different at the 5% level.

^c^ Statistically significant difference between all infant formula and human milk samples if *p* value < 0.05.

## 4 Discussion

### 4.1 Copper

Copper levels in both human milk and infant formula samples were below the A.S.P.E.N recommendations (Vanek et al. 2012). This raises concerns about the inadequate levels observed in our study and their potential impact on overall infant development. Low copper concentration may cause significant clinical outcomes, including anemia, thrombocytopenia, neutropenia, and osteoporosis (Bhatia et al. 2013). These findings underscore the critical need to fortify human milk (Radmacher and Adamkin 2017) to ensure that preterm infants receive adequate copper intake according to established recommendations. The mean concentration of copper in human milk reported in our study (0.505 μg/mL) closely aligns with findings from a study conducted in Korea (0.506 μg/mL) (Kim et al. 2012) and a study in China (0.573 μg/mL) (Lin et al. 2022). Conversely, 2 studies in Japan reported lower copper concentrations with a mean of (0.83 μg/mL) (Ohtake and Tamura 1993) and (0.35 μg/mL) (Yamawaki et al. 2005). Regarding infant formula, our results indicated a higher copper concentration (3.62 μg/g) than studies conducted in Japan (0.437 μg/g) (Higashi et al. 1988), Canary Islands (0.513 μg/g) (Rodriguez Rodriguez et al. 2000), and North Carolina (0.340 μg/g) (Eaves et al. 2023) The comparative analysis of copper concentrations in human milk and infant formula revealed similar levels in human milk (0.505 μg/mL) and infant formula (0.469 μg/mL). Conversely, a study conducted in Japan indicated that the copper content in infant formula was 0.05 μg/mL, which is approximately 6.2 times lower than the concentration of 0.31 μg/mL found in human milk (Higashi et al. 1982). However, these findings contrast with another study conducted in Spain, which reported that formula milk had a copper concentration of (0.39 μg/mL), approximately 1.26 times higher than that found in human milk (Rodriguez Rodriguez et al. 2000).

### 4.2 Zinc

Our study found that zinc levels in human milk and infant formulas were below the A.S.P.E.N. recommendations (Vanek et al. 2012), which call for the use of milk fortification. Preterm neonates are particularly vulnerable to zinc deficiency due to factors such as low body stores, increased urinary losses, inadequate intake, immature gastrointestinal function, and heightened demand, which can lead to complications such as dermatitis, growth impairment, and an increased risk of chronic lung disease and necrotizing enterocolitis (Terrin et al. 2015). In our study, the zinc concentration in human milk (2.317 μg/mL) was similar to that found in studies conducted in Switzerland (2.4 μg/mL) (Sabatier et al. 2019), Korea (2.451 μg/mL at 1 month) (Han et al. 2011), and Iran (2.95 μg/mL) (Khaghani et al. 2010). However, our results were lower than those reported in Brazil (4.15 μg/mL) (Trinta et al. 2020), Canada (4.13 μg/mL) (Mendelson et al. 1982), and South Korea, where zinc concentrations ranged from 7.8 μg/mL at week 1 to 6.6 μg/mL at week 12 (Kim et al. 2012). Furthermore, the zinc concentration in infant formulas in our study (26.74 μg/g) falls within the ranges found in studies in Saudi Arabia (4.2–139.7 μg/g) (Alfaris et al. 2022), Poland (32.2–101.0 μg/g) (Dobrzynska et al. 2021), and Egypt (22.46–87.3 μg/g) (Ghuniem et al. 2019). However, it was lower than those reported in studies from Spain (32.12 μg/g) (Moreno‐Rojas et al. 2014) and Poland (41.2 μg/g) (Lesniewicz et al. 2010). Moreover, our study found that the zinc concentration in reconstituted infant formulas was 1.5 times higher than in human milk. This finding aligns with similar studies, including an Iranian study that reported a 1.35‐fold increase (Khaghani et al. 2010), a study conducted in Korea that observed a 1.79‐fold increase in zinc content in one month (Han et al. 2011), and a study in Brazil that found nearly double the zinc content in infant formulas (Fioravanti et al. 2020).

### 4.3 Iron

Iron levels in both human milk and infant formula samples were below the A.S.P.E.N recommendations (Vanek et al. 2012). Preterm infants are particularly vulnerable due to diminished iron stores accrued during the third trimester, accelerating the depletion of these stores during early postnatal growth, and complications such as chronic gastrointestinal hemorrhage (Rao and Georgieff 2009). Effectively, between 25% and 85% of preterm infants exhibit signs of iron deficiency within the first 6 months of life, with the risk being highest among those born at earlier gestational ages or with lower birth weights (Rao and Georgieff 2009). Iron supplementation is thus recommended for this population (Rao and Georgieff 2009), since deficiency is associated with a range of adverse outcomes, including impaired physical and neurological growth, gastrointestinal disturbances, thyroid dysfunction, compromised immune function, and difficulty regulating body temperature (Rao and Georgieff 2009). Analysis of iron content in infant formulas revealed notable differences across studies. In our study, the iron concentration in infant formula was 52.88 μg/g, which is significantly higher than the value reported in a study conducted in Poland (1.80 μg/g) (Lesniewicz et al. 2010). Comparatively, iron concentrations in infant formulas from Spain and Turkey ranged from 41.4 to 97.4 μg/g (Yebra et al. 2004) and from 1.02 to 67.5 μg/g (Saracoglu et al. 2007), respectively, aligning more closely with the values observed in our study. Additionally, a study conducted in Saudi Arabia reported a wider range of iron levels in infant formula (1–117.3 μg/g) (Alfaris et al. 2022), with the upper limit exceeding the concentrations observed in our study. The analysis of human milk in our study revealed a mean iron concentration (0.917 μg/mL), which is higher than the values observed in studies conducted in Brazil (0.66 μg/mL) (Goes et al. 2002) and Poland (0.33 μg/mL) (Bzikowska‐Jura et al. 2021), but within the range found in a study in Italy (0.23–1.84 μg/mL) (Bocca et al. 2000). Moreover, our study found that the iron concentration in reconstituted infant formulas was approximately 7.5 times higher than in human milk (6.857 vs. 0.917 μg/mL). Similarly, a Polish study reported a comparable trend, with iron levels in infant formulas being 3.1 times higher than in human milk (6.9 vs. 2.2 μg/mL) (Purkiewicz et al. 2024). Several factors may influence iron concentration comparisons between infant formulas and human milk, including deliberate formula fortification policies (mandated since 1969) to compensate for lower bioavailability (10%–12% from formula vs. 50%–70% from human milk) (El Safy et al. 2016) (Saarinen and Siimes 1979), and population‐specific variations in maternal milk composition including ethnicity and geographic location significantly affect human milk iron content, with variations ranging from 0.81 mg/L in Chinese mothers to 1.11 mg/L in Indian mothers (Fatima et al. 2022), whereas human milk iron concentration remains largely independent of maternal iron status (Friel et al. 2018).

### 4.4 Manganese

Our study found that manganese levels were above A.S.P.E.N recommendations (Vanek et al. 2012) for 86% of infant formula samples and below those recommendations for 34% of human milk samples. The International Expert Group (IEG) has warned against elevated manganese levels since both preterm and term infants have limited capacity to excrete manganese efficiently, given its classification among six emerging developmental neurotoxicants. Notably, early neurotoxic exposure may lead to cognitive deficits and behavioral alterations that remain latent until school age or later developmental stages (Grandjean and Landrigan 2014). Manganese concentrations observed in our study for human milk (0.005 μg/mL) are consistent with previously reported findings in Italy (0.006 μg/mL) (Prohaska et al. 2000) and in the United States (0.0049 μg/mL) (Stastny et al. 1984), whereas concentrations in our infant formula samples (0.34 μg/g) were lower than those reported in Brazil (3.17 μg/g) (Almeida et al. 2022). Our study revealed that manganese levels in infant formula (0.115 μg/mL) were approximately 23 times higher than in human milk (0.005 μg/mL). This significant disparity is consistent with research in Sweden (Ljung et al. 2011), France (Frisbie et al. 2019) and the EU (Pandelova et al. 2012) that reported higher manganese concentrations in infant formula, ranging from 100 to 1000 times that of human milk.

### 4.5 Mineral Content in Infant Formula as Tested in the Laboratory

Our study found that preterm infant formulas had nonsignificant higher mineral concentrations of zinc, iron, and copper than term formulas, whereas term formulas had significantly higher manganese concentrations. Our findings align with a study conducted in Spain that reported similar results (Sola‐Larrañaga and Navarro‐Blasco 2006). However, this is in contrast with another study in Spain which indicated that term infant formulas contained higher concentrations of zinc and iron compared to preterm formulas (Moreno‐Rojas et al. 2014). According to A.S.P.E.N. daily nutrient requirements (Vanek et al. 2012), preterm infants have higher daily mineral requirements for copper, zinc, and manganese, whereas the iron requirements are the same for both preterm and term infants. Although the Codex standard for infant formula does not specify requirements for each type of formula, it is essential that infant formulas meet the nutritional needs of infants by providing adequate levels of each required mineral (Washington, D.U.S.G.P.O. 2024).

### 4.6 Mineral Content in Infant Formula: Laboratory Versus Label

Although the 3 minerals Cu, Zn, and Mn met the Codex standard (WHO, F 2007) for infant formula, Iron met the minimum recommended levels specified by Codex and exceeded the maximum level of EU regulation (European Union 2016) in 10% of the samples. Furthermore, some factors should be considered when evaluating the significance of excess iron, including iron bioavailability. Bioavailability from dairy and soy‐based infant formulas is lower than that of human milk by roughly 40% and 84%, respectively (Strzalkowski et al. 2023). This may explain why the Codex standard (WHO, F 2007) does not specify maximum levels of iron in infant formula, as there is limited evidence to support such requirements. Discrepancies were observed between reported levels on labels and tested levels, with statistically significant differences between lab and label values for Cu (*p* value = 0.0039) and Zn (*p* value = 0.000). When manufacturing infant formulas, several factors must be considered. Each formula product must undergo individual testing and labeling, and labels cannot be copied between products; even if they are produced by the same manufacturer (Washington, D.U.S.G.P.O. 2024). The FDA requires verification to ensure that the contents of the product align with the claims presented on the label (Washington, D.U.S.G.P.O. 2024). Additionally, infant formula manufacturers typically test nutrient content using batch sampling instead of testing each container individually. This approach can lead to variations in nutrient levels between containers within the same batch due to mixing inconsistencies and the manufacturing process (Washington, D.U.S.G.P.O. 2024).

### 4.7 Comparison of Mineral Content in Human Milk and Infant Formula

Our results showed that reconstituted infant formulas had significantly higher levels of zinc, iron, and manganese than human milk, except for copper, where similar levels were reported in both human milk and infant formulas. It is noteworthy to highlight that despite the higher levels reported in infant formula, all human milk and infant formula samples were below the recommendations for zinc and iron. Furthermore, the levels of Mn in 86% of infant formula samples exceeded dietary recommendations. Despite human milk having generally lower mineral concentrations, it maintains its position as the gold standard in infant nutrition, particularly for preterm infants (Eidelman 2012; Victora et al. 2016). This superiority derives from its complex matrix of bioactive substances, including specialized proteins, hormones, enzymes, and growth factors that enhance nutrient absorption through mechanisms absent in formula preparations (Lonnerdal 2017; Koletzko 2016; Hernell et al. 2016; Bode 2015; McGuire and McGuire 2017; Bode et al. 2014). Recent studies have established that human milk plays a protective role against significant complications in preterm infants, particularly by reducing the incidence of necrotizing enterocolitis and sepsis (Maffei and Schanler 2017; ESPGHAN Committee on Nutrition et al. 2013; Corpeleijn et al. 2012; Schanler et al. 2005; Sullivan et al. 2010). Recent research advocates for the fortification of human milk once the feeding volume reaches 50–80 mL/kg/day (Arslanoglu et al. 2013). This evidence‐based strategy has positioned human milk feeding as a fundamental right for preterm infants, establishing a clear hierarchy: maternal milk is deemed the optimal choice, followed by donor milk in cases of insufficient maternal supply (Arslanoglu et al. 2013).

## 5 Limitations

Several limitations were acknowledged in this study. First, the disparity in sample sizes between preterm (*n* = 8) and term (0–6 or 12 months) (*n* = 34) formulas, attributed to limited commercial availability of preterm‐specific products, may compromise the generalizability of the findings for this vulnerable population even though the selected type of term formula can be consumed by both term and preterm infants. Second, while our study's primary objective was to assess baseline mineral composition to determine the potential need for fortification, the exclusion of human milk fortifiers from our analytical framework represents a gap that requires future investigation, as these products constitute essential nutritional interventions for preterm infants. Third, the influence of maternal diet and lifestyle on the mineral composition of human milk was not examined, as this was beyond the scope of the current study. Fourth, our study omitted comprehensive analysis of milk bioactive compounds like A1/A2 β‐casein variants and infant biomarkers like inflammatory factors across heterogeneous infant populations and demographic subgroup analyses, as these were beyond our primary research objectives but are an interesting perspective. Finally, the cross‐sectional design of this study limits our ability to capture longitudinal changes in mineral composition and assess their clinical impact over time. As a result, causal relationships and long‐term health implications could not be determined.

## 6 Conclusion

This study assessed the mineral content of human milk and infant formula and compared mineral levels to established nutritional recommendations for preterm infants. The findings underscore the necessity of ensuring adequate levels of copper, zinc, manganese, and iron to support optimal growth and development in this vulnerable population. Notably, the study identifies significant deficiencies in copper, zinc, and iron in both human milk and infant formula, which call for further research to address these gaps and their potential health implications. Additionally, the elevated manganese levels observed in infant formula, which exceed the recommended nutrient requirements for preterm infants, warrant further investigation to assess their potential toxicity. These findings underscore the importance of routine monitoring of both the mineral content in infant formulas and the accuracy of nutrition facts labels to ensure they align with actual nutrient levels and support the nutritional adequacy of products intended for this vulnerable population. Additionally, further investigation is warranted with expanded sample sizes, prolonged monitoring durations, and assessment across various infant developmental stages to validate the clinical benefits of infant formula. The study emphasizes the need for further research into the concentrations of other essential minerals in human milk and infant formula and their compliance with established nutritional recommendations. It would also be interesting to study the associations between maternal factors, such as diet and lifestyle, and the content of minerals in human milk. It is also important to investigate the impact of different feeding patterns such as exclusive breastfeeding, mixed feeding, and exclusive formula feeding on infant health outcomes.

## Author Contributions

**Zainab Haji:** data curation (equal), formal analysis (equal), visualization (equal), writing – original draft (equal), writing – review and editing (equal). **Rana Mohammed:** data curation (equal), formal analysis (equal), visualization (equal), writing – original draft (equal), writing – review and editing (equal). **Hanan Fardan:** data curation (equal), formal analysis (equal), visualization (equal), writing – original draft (equal), writing – review and editing (equal). **Najmeh Vatankhah:** data curation (equal), visualization (equal), writing – original draft (equal), writing – review and editing (equal). **Shrooq Ismail:** data curation (equal), methodology (equal), writing – original draft (equal). **Maya Bassil:** funding acquisition (equal), investigation (equal), project administration (equal), resources (equal), supervision (equal), writing – review and editing (equal). **Zumin Shi:** investigation (equal), methodology (equal), software (equal), validation (equal), writing – review and editing (equal). **Mohammad Ibrahim Ahmad Ibrahim:** data curation (equal), methodology (equal), resources (equal), writing – original draft (equal). **Grace Attieh:** project administration (equal), resources (equal), software (equal), supervision (equal), writing – original draft (equal). **Nader Al‐Dewik:** funding acquisition (equal), project administration (equal), resources (equal), writing – review and editing (equal). **Layal Karam:** conceptualization (equal), data curation (equal), formal analysis (equal), funding acquisition (equal), investigation (equal), methodology (equal), project administration (equal), resources (equal), supervision (equal), validation (equal), visualization (equal), writing – original draft (equal), writing – review and editing (equal).

## Ethics Statement

The study had received Institutional Review Board (IRB) approval from Qatar University (QU IRB #: QU‐IRB 133/2024‐EM) and HMC Medical Research Center (HMC IRB #: MRC‐01‐22‐231) and consent forms were obtained from all participants. It received as well approval from the Qatar University Institutional Biosafety Committee (QU IBC #: QU‐IBC‐006/2025).

## Conflicts of Interest

The authors declare no conflicts of interest.

## Data Availability

Data is available upon request.
